# Got GAS? Ease the Bloat with Real-Time Whole-Genome Sequencing

**DOI:** 10.1017/ice.2018.73

**Published:** 2018-04-02

**Authors:** Emil P. Lesho, Erik Snesrud, Melissa Bronstein, Margaret Pettis, Ana Ong, Rosslyn Maybank, Yoon Kwak, Anthony Jones, Kelly Vore, Patrick McGann, Mary Hinkle

**Affiliations:** 1 Infectious Diseases Unit, Rochester Regional Health, Rochester, New York; 2 Multidrug-resistant Organism Repository and Surveillance Network, Walter Reed Army Institute of Research, Silver Spring, Maryland; 3 Quality Safety Institute, Rochester Regional Health, Rochester, New York; 4 Infection Prevention, Rochester Regional Health, Rochester, New York.

*To the Editor—*Annually, more than 10,000 patients in the United States acquire an infection caused by invasive group A *Streptococcus* (GAS). The fatality rate of this illness is 11.7%, and many infections are transmitted person to person.^1^ ^,^ ^2^ Outbreak investigations of postsurgical group A *Streptococcus* (GAS) infections can substantially disrupt surgical throughput if staff require furloughing, and they can be extremely labor intensive when surgeons practice at multiple facilities.^3^ One benefit that has received little attention is the labor-saving potential that whole-genome sequencing (WGS) offers infection preventionists (IPs) when the turnaround time is sufficiently rapid to inform investigations and mitigation efforts.^4^ Here, we highlight an outbreak involving 22 surgical staff, several of whom practice at multiple facilities that often care for the same patients within a regional care network.

On day 0, patient A underwent a procedure at community hospital X, performed by surgeon I who also practices at referral hospital Y (Table 1). On day 5, patient A developed an invasive GAS surgical wound infection while at hospital X. On day 7, patient B underwent a procedure performed by surgeon II at hospital X. On day 8, patient B developed a complication requiring escalation of care to hospital Y for follow-up surgery, again performed by surgeon II. On day 13, GAS was isolated from the surgical wound of patient B while at hospital Y. The 2 GAS isolates were sent for WGS, using methods described previously.^4^ ^,^ ^5^ Simultaneously, IP staff initiated a retrospective review of all laboratory results beginning 6 months prior to the first surgery. Involved surgical staff at all facilities were contacted to have their throats and groins swabbed. Mitigation planning was begun in case staff furloughing would be required pending decolonization.

**TABLE 1:** Potential Impact of an Outbreak Investigation for Surgical Site Infection due to Group A *Streptococcus*

Day and Event	Patient / Hospital	Surgeon / No. of Support Staff
Day 0, Surgical procedure	Patient A / Hospital X	Surgeon I / 1 Staff
Day 5, GAS infection	Patient A / Hospital X	
Day 7, Surgical procedure	Patient B / Hospital X	Surgeon II / 9 Staff
Day 8, Surgical procedure	Patient B / Hospital Y	Surgeon II / 10 Staff
Day 13, GAS infection	Patient B / Hospital Y	
		Total of 22 Possible Carriers
Costs	Conventional approach	Real-time sequencing approach
**Direct**		
Laboratory	$175.00	$80.00
Infection preventionist labor^a^	10 RN+hours=$500.00	$0.00
Employee health labor^a^	8 RN+hours=$400.00	$0.00
**Indirect**		
Lost revenue due to time surgical staff not operating due to specimen collection or on furlough if screened positive and awaiting genotyping and/or decolonization	Potentially $1,000–100,000 depending on number of staff involved and time line	$0.00

a RN+, registered nurse at 90% effort with Advanced Practitioner or Physician Oversight at 10% effort.

The core genome sequences of the 2 isolates differed by ~40,000 nucleotide changes, indicating that they were genetically unrelated.^5^

The WGS results were available within a week, before all staff had been swabbed and before any culture results of those that had been swabbed were available. On other occasions, results have been available in <50 hours.^4^ For this event, WGS permitted earlier termination of the investigation and faster resumption to full surgical capacity, saving time, labor, and money (Table 1). The costs in Table 1 were calculated based on material and labor costs in this region^6^ for screening all involved operating room staff (n=22). If WGS had determined that the isolates were related, the cost would have been $80.00 more for the WGS approach compared to the conventional approach (not using WGS). When WGS revealed that the isolates were unrelated, the cost savings were substantial because surgical throughput was not slowed or disrupted, and IPs were able to devote their time and efforts to other issues. Currently, WGS has become faster, less expensive, and more informative than pulsed-field gel electrophoresis (PFGE). Furthermore, PFGE has been suggested to lead to erroneous conclusions regarding genetic relatedness among strains.^7^ However, such timely feedback is not yet available to most hospitals; thus, IPs, surgical facilities, and patients would benefit from wider access to real-time, genome-based support.
